# Diseases and Treatment of the Dental Pulp

**Published:** 1870-08

**Authors:** F. Y. Clark

**Affiliations:** Savannah, Georgia.


AMERICAN JOURNAL

OF

DENTAL SCIENCE.

Vol. IV. THIRD SERIES--AUGUST, 1870. No. 4.

### ARTICLE I.

### *Diseases and Treatment of the Dental Pulp.*

By F. Y. Clark. D.D.S. of Savannah, Georgia.

Read before the Southern Dental Association.

There is no operation that the Dental practitioner is called on to perform of more importance to himself and patient than the restoration of a tooth to health and usefulness, after the exposure of its lining membrane. When we turn back in Dental History some twenty years, or more, and view the treatment of exposed pulps, in comparison with the treatment now we may reasonably open our eyes in wonder and admiration. In those days it would have been considered absurd, if not quackery, to talk about and advocate the practice now in vogue, and we sometimes think could some of our old "sober-sided" predecessors but awake from their sleep, as did Rip Van Winkle, that they would find themselves as much out of place in the ranks of our profession as did this good old gentlemen amongst his former friends of the Hudson.

With these preparatory remarks, we will now consider the exposure and treatment of the dental pulp in its various conditions, and give our manner of treatment; but in doing so, we hope we will not be considered as claiming any better practice, or more success, than many others; but merely as explaining our mode and giving our experience that others may compare it with their own, and adopt, or reject it, as their own good judgement will indicate.

Where there is simile exposure of the dental pulp, caused by accident in excavating or in any other case, no matter how caused—provided there is, or has been, no inflammation—we consider the best course in all such cases, to attempt to save the vitality of the tooth and induce, if possible, ossific deposit. In doing this, a great deal of care and nice manipulation is necesary in removing decay and detached dentine from over and around the pulp. Should any detached particles be forced into the canal, or on the pulp, so as to irritate, inflammation and failure is sure to follow. A drop or two of blood will do no harm, but sometimes will prove beneficial. After all loose decay is removed, the cavity should be flooded with some one of the essential oils—we generally use sulphuric ether or spirits of camphor, particularly where there is no pain. After this creosote should be used, allowing it to remain two or three minutes; it is sufficiently escharotic, and its antiseptic properties are about what is wanted. As a general rule, we do not approve of temporary fillings in this condition of the pulp; we think a temporary filling indifferently put in is much more liable to cause trouble than a permanent one carefully introduced. If care is taken, a good permanent filling may be put in with as much, or more, safety than a temporary one. The gold should be packed carefully over and around the exposed part, so as not to produce pressure on the membrane. In doing this, the *modus operandi* will have to be varied according to the case. What will apply to one will not to another. So as pressure on the exposed point is avoided, is all that is necessary. A way to do this will suggest itself to the mind of every intelligent operator. If the cavity is one that can be seen, and there is a fair chance to operate, a good permanent filling may be introduced without much fear of trouble; but where a full view of every part can not be had, it is no doubt better to fill the floor with os-artificial, and then, when this hardens, with gold.

The second condition of an exposed pulp is one that has been exposed for some time. The tooth may have ached at different periods, and may be in an aching condition at the time of treatment. The least pressure or contact with cold fluids will produce pain. The exposure and decay is more extensive than the one just described; but the periosteum is not implicated. In this class of exposure, we consider our treatment almost certain, and do not look for trouble with one case in twenty. We proceed first to excavate around the margin of the cavity, removing as much of the decay as is practicable without pain, shapeing the walls of the cavity so that it will retain the application. In placing our preparation on the nerve, which is composed of the usual arsenious paste, our aim is to avoid pain and the action of the arsenic on the dentine. In order to do this, we take a plug of wax, giving it a cone shape and making a hole through its center large enough to admit the point of our ordinary excavator; we then pass a small bit of common wrapping cord through the hole, allowing the end on the cone side of the wax, which is to go into the cavitiy, to project a very little beyond the wax, so that in placing it into the cavity the end of the cord, on which there is a speck of paste, will come in contact with the pulp, first with a chisel-like instrument slit from the point up the center half an inch, so as to admit the outside end of the cord, the wax is placed in position, the cord withdrawn and the wax on the surface flowed by a warm instrument. Thus it will be seen the arsenious paste is brought in direct contact with the nerve, and when the cord is withdrawn there is a small vacuum, thereby avoiding the pain as is usually produced by pressure. This little auxiliary, in the hands of a neat operator, will prove of great advantage; but with a bungler it will be no better than cotton and varnish or other things generally used.

We prefer making the application to the pulp in the morning, allowing it to remain until the next day afternoon, from twenty-four to thirty hours.

On removing the wax, the partition over each root should be broken down and excavated away, so as to give a full view of the interior, if possible. The pulp should not be mangled up, as is generally the case, for then it will have to be taken away in fragments, giving considerable pain, and frequently considerable trouble. If it is in a flabby state, so that it can be wiggled about without any or much pain, it should then be removed at once. In this consideration, if proper instruments and care is used, it will generally come away whole, leaving the root at the foramen: but sometimes we do not find this condition of affairs. We may be obliged to inflict considerable pain, the pulp may come away in fragments, and there may be considerable bleeding; but on completing the removal, if the pain and bleeding disappear and there is no other unfavorable symptom, we do not hesitate to fill at once, being convinced by experience that it is much better to do so than to delay until a second or third setting. If, however, owing to the constitution or temperament of the patient or to some unforseen cause, as is now and then the case, vascular excitement is produced and periosteal inflammation threatens, we should not be at a loss to know what to do, or in the least despair of success. The first stage of trouble is the time for preventative treatment, and if remedies are properly and timely applied, periostitis or an alveolar abscess may nearly always be prevented. If the system is disordered or in an inflamed condition, a cathartic along with the application of iodine and aconite to the gum adjacent to the affected tooth; two parts of comp. tinct. of iodine to one of aconite. This should be used with a little brush, keeping the mouth open until absorption is finished. If one or two applications of this fails to give relief, and there is considerable inflammation, and apparent engorgement of the surrounding vessels, their thorough depletion over the affected part will, in most cases, give relief and allay the excitement. We generally deplete by making a deep incision in the gum, immediately over the apex of the root, and when the bleeding has fully subsided, insert a pledget of cotton into the opening, this will act as a counter irritant, and, in the course of a few days will come away. If this fails to relieve the trouble, we either trephine the alveolus or turn our attention to the encouragement of an abscess, or extract the tooth.

We now come to another or third condition of the pulp, which gives more trouble, on account of its not being understood, than any other state in which it is found. We allude to that class of teeth where the pulp is devitalized and where a discharge has been going on for months and perhaps years; where there is, or never has been, a fistulous opening. There are several varieties of this class—some without any apparent discharge whatever; others, again, whose discharge is extensive and offensive; but as the same treatment will ap ply to all, we will therefore treat of them under one head. Years ago, with this class, we with many others were in the habit of feeling our way along the best we could, with nothing reliable or certain to guide us. We knew we had much better success by a mild course of treatment than by a more heroic one, but did not know why. The age, temperament and habits of the patient, along with a cheerful willingness to go halves in the risk, were our most reliable assistants; but now we think we understand them better, and believe we know the cause of the trouble. In this class of teeth the roots are encumbered with debris and morbid matter, and it is usually the course to attempt the removal of this debris by a barbed instrument at the first sitting, working the instrument up and down, pump-like, thereby either choking up the foramen and causing the discharge to hunt another outlet, or forcing particles or gases through, creating vascular excitement, periostitis and alveolar abscess, or the loss of the tooth. Again; the foramen, in this condition of tooth, owing to a current of corrosive matter passing through it, is somewhat enlarged, and it is not unfrequent that the fine hair-sized broaches used, are passed through, irritating the parts beyond which are generally in a state to inflame on the slightest provocation. Now, as the aggravation is produced by closing up the natural opening for the pus in the process of barbing out the root, or by forcing an instrument or gases through, irritating the parts above the apex, it is clear to see if this is prevented no trouble will ensue.

The first attack upon a tooth in this condition, after making a clear opening to the mouth of the pulp canal, should be made with injections of warm water, followed with a mild preparation of carbolic acid, compound tinc. of iodine, glycerine and water. The following is the formula which we use; carbolic acid, gtt. 8; compound tinct. iodine, gtt. xv; glycerine, ℥ vij; water, ℥ vj. After a free use of this, the tooth should be packed with cotton for a day or two, then washed out with this preparation again, and the root filled with a thread moistened in creosote, and a temporary filling of gutta-percha, or Hill's stopping. It should be allowed to remain in this state for several days. If at the end of that time there is no offensive odor, the root may be barbed out and filled without fear of further trouble; but if, as is now and then the case, creosote is not strong enough or sufficiently antiseptic to remove all odor, it may be increased in strength and action by the addition of a little carbolic acid and it may be increased in strength at will. It is best in this condition of teeth to keep up the antiseptic properties of the creosote for some time, and therefore it should be introduced with the filling.

We now come to another class of teeth with devitalized pulps, which differ from the last by having an outlet through the alveolus and the gum, through which the morbid matter passes, instead of through the root, as in the ones just treated. The main point in the treatment of this class consists in breaking up the disorganization at the end of the root, and curing the fistula. This can generally be accomplished at one sitting, without fear or risk. A small hair-sized nerve instrument should be passed through the foramen, until there is a slight indication of its touching the parts beyond. A barbed instrument, with a little cotton wrapped around it, and dipped in creosote, should then be introduced, and moved up and down until the creosote is seen to come out on the surface of the gum, through the fistula.

---

